# Immune cell concentrations among the primary tumor microenvironment in colorectal cancer patients predicted by clinicopathologic characteristics and blood indexes

**DOI:** 10.1186/s40425-019-0656-3

**Published:** 2019-07-12

**Authors:** Guifang Guo, Yixing Wang, Yixin Zhou, Qi Quan, Yijun Zhang, Haohua Wang, Bei Zhang, Liangping Xia

**Affiliations:** 1 VIP Department, Sun Yat-sen University Cancer Center, 651 Dongfeng Road East, Guangzhou, 510060 People’s Republic of China; 2 State Key Laboratory of Oncology in South China, Sun Yat-sen University Cancer Center, 651 Dongfeng Road East, Guangzhou, 510060 People’s Republic of China; 3 Collaborative Innovation Center for Cancer Medicine, Sun Yat-sen University Cancer Center, 651 Dongfeng Road East, Guangzhou, 510060 People’s Republic of China; 4 Guangdong Key Laboratory of Nasopharyngeal Carcinoma Diagnosis and Therapy, Sun Yat-sen University Cancer Center, 651 Dongfeng Road East, Guangzhou, 510060 People’s Republic of China

**Keywords:** Tumor microenvironment, Immune cells, Immunoscore, Colorectal cancer, Blood indexes

## Abstract

**Background:**

Immune cells play a key role in cancer progression and treatment. It is unclear whether the clinicopathologic characteristics and blood indexes of colorectal cancer (CRC) patients could predict immune cell concentrations in the tumor microenvironment.

**Methods:**

CRC patients with detailed data and tumor tissue who visited Sun Yat-sen University Cancer Center between April 1, 2004, and September 1, 2017, were enrolled. The densities of CD3+ and CD8+ T cells examined by immunohistochemistry in both the core of the tumor (CT) and the invasive margin (IM) were summed as the Immunoscore. The relationships between the Immunoscore and clinicopathologic characteristics and blood indexes, including tumor biomarkers (carcinoembryonic antigen (CEA) and carbohydrate antigen 19–9 (CA 19–9)), inflammatory markers (lactate dehydrogenase (LDH), C-reactive protein (CRP), albumin (ALB), neutrophils, lymphocytes, monocytes, platelets, NLR (neutrophil-to-lymphocyte ratio), PLR (platelet-to-lymphocyte ratio) and LMR (lymphocyte-to-monocyte ratio)) and lipid metabolism markers (cholesterol (CHO), triglyceride (TG), high-density lipoprotein (HDL), low-density lipoprotein (LDL), apolipoprotein A1 (ApoA1), and apolipoprotein B (ApoB)), were analyzed using SPSS.

**Results:**

Older patients had lower CD3+ and CD8+ T cell expression in the IM and a lower Immunoscore than did younger patients. CD8+ T cell expression in the IM and the Immunoscore were lower in right-side tumors than in left-sided tumors. High CD8+ T cell expression in the CT was found in the T4 stage group. The higher the CEA level in the blood, the fewer CD8+ T cells were in the CT. Either fewer monocytes or a higher LMR in the blood, the larger number of CD3+ T cells in the CT. The more ApoA1 was in the blood, the more CD3+ T cells were in both the CT and the IM.

**Conclusion:**

Age, T stage, tumor location, CEA, monocytes, LMR and ApoA1 could reflect immune cells infiltrating the tumor microenvironment of CRC.

## Introduction

Colorectal cancer (CRC) is one of the main causes of cancer-related deaths around the world. The prognosis of patients relies on histopathological criteria of tumor invasion according to the American Joint Committee on Cancer (AJCC) and Union for International Cancer Control (UICC) TNM classification system and on features of tumor cell differentiation [1, 2]. This approach provides useful but incomplete information to predict prognosis. There has been increasing interest in predicting CRC prognosis, focusing on tumor cells, molecular pathways, mutation status, inflammation and immune cell infiltration [3, 4].

Human immunity has a complex and multifaceted role in cancer, affecting all aspects of the disease, from tumorigenesis to treatment [5]. Immune cells can act both as suppressors of tumor initiation and progression and as promoters of proliferation, infiltration and metastasis [6]. In the tumor microenvironment, various immune cells, both innate immune cells and adaptive immune cells, have been reported in all tumor types depending on the tumor origin, location, and individual characteristics. The Immunoscore was confirmed to predict clinical outcome in patients with early- [7] and advanced [8]-stage CRC. The Immunoscore, a derived immune score, is a scoring system that summarizes the density of CD3+ and CD8+ T cell effectors within the tumor and its invasive margin. It has been suggested that the use of the Immunoscore in combination with the AJCC/UICC stage could lead to a better determination of tumor prognosis [3].

Blood indexes, such as tumor markers, systemic inflammation and lipid metabolism, are also correlated with cancer prognosis. There is a growing consensus that inflammation is involved in the development of malignancy and that an ongoing systemic inflammatory response is associated with a worse prognosis [9]. These factors include lactate dehydrogenase (LDH) levels; C-reactive protein (CRP) levels; albumin (ALB) levels; the numbers of neutrophils, lymphocytes, monocytes, and platelets; the NLR (neutrophil-to-lymphocyte ratio); the PLR (platelet-to-lymphocyte ratio); and the LMR (lymphocyte-to-monocyte ratio). The tumor markers in CRC, carcinoembryonic antigen (CEA) [10] and carbohydrate antigen 19–9 (CA 19–9) [11], can predict the prognosis of CRC. Additionally, several studies have demonstrated the importance of lipid metabolism regulation in the promotion of migration [12], invasion [13], and angiogenesis [14]. Lipid metabolism is associated with cancer survival and has been proposed as a prognostic marker [15].

In light of these recent findings, both the immune state and the above blood indexes could impact prognosis. Do they have any relationship? The present study aimed to investigate the association between CD3+ and CD8+ T immune cells in the tumor microenvironment and clinicopathologic characteristics and blood indexes, including tumor markers, inflammatory indictors and lipid metabolism factors, for patients with CRC. We hope to offer evidence to monitor the immunity status of the CRC microenvironment by basic indexes.

## Materials and methods

### Study population

A retrospective study was conducted in patients with CRC at Sun Yat-sen University Cancer Center between April 1, 2004, and September 1, 2017. All patients had histologically proven CRC at the primary tumor site, and all cases of CRC were adenocarcinomas. Additionally, patients had not previously taken anti-inflammatory medicine or immunosuppressive therapy, including recent steroid exposure, or had chronic inflammatory disease, including autoimmune disease and infections. Basic characteristic information for all the patients was collected. The study was approved by the Institutional Review Board and Ethics Committee at Sun Yat-sen University Cancer Center.

### Laboratory measurements of blood indexes

Many biomarkers, including tumor biomarkers, inflammatory markers and lipid metabolism markers, were examined in our study. CEA and CA 19–9 were included as tumor markers. LDH, CRP, ALB, neutrophils, lymphocytes, monocytes, platelets, NLR, PLR and LMR were included as inflammatory markers. Cholesterol (CHO), triglyceride (TG), high-density lipoprotein (HDL), low-density lipoprotein (LDL), apolipoprotein A1 (ApoA1), and apolipoprotein B (ApoB) were included as lipid metabolism markers. The biomarkers included in our study were measured in each included patient before surgery or biopsy within 2 weeks using laboratory devices in our cancer center. LDH, CRP, ALB, CHO, TG, HDL, LDL, ApoA1, and ApoB were included in a biochemical test performed using a Hitachi Automatic Analyzer 7600–020 (Tokyo, Japan), and CEA and CA 19–9 in serum tumor marker tests were measured using a Roche Elecsys 2010 Chemistry Analyzer (Basel, Switzerland). Neutrophils, lymphocytes, monocytes and platelets were measured by routine blood examination (XE-5000TM Automated Hematology System). The normal ranges of CEA, CA 19–9, LDH, CRP, ALB, CHO, TG, HDL, LDL, ApoA1 and ApoB levels in blood were 0–5 ng/ml, 0–35 U/ml, 120–250 U/L, 0–3 mg/L, 40–55 g/L, 3.1–5.69 mmol/L, 0.2–1.7 mmol/L, 1.16–1.42 mmol/L, 2.2–3.1 mmol/L, 1.2–1.6 g/L, and 0.6–1.1 g/L, respectively. The levels of NLR, PLR and LMR, as specific values, did not have a standard normal range.

### Immunohistochemical staining

Pathologic slides prepared with surgical or biopsy specimens preserved in paraffin blocks were stained with monoclonal antibodies against CD3 (Cell Signaling Technology, United States; Catalog No. 85016S) and CD8 (Cell Signaling Technology, United States; Catalog No. 85336S). Stained sections from representative areas of the core of the tumor (CT) and invasive margin (IM) were scanned using an Olympus digital slide scanner. Computer-assisted calculations of the density of CD3+ and CD8+ T cells in both the CT and the IM of the tumor were conducted using ImageJ v1.48, a public domain, Java-based image processing program developed at the NIH (National Institutes of Health, Bethesda, MD, USA), as described by Galon et al. [3]. Two independent pathologists who were blinded to the patients’ clinical information participated in the analysis to recognize the location of the CT/IM. Immunoscore evaluations were performed based on the densities of CD3+ and CD8+ T cells in both the CT and IM regions by the cut-off of the median of each index (CD3+ T cells in the CT and IM, CD8+ T cells in the CT and IM). A low value was scored as 0, whereas a high value was scored as 1. The sum of all scores was calculated as the final Immunoscore. For example, I0 refers to a tumor with low densities of CD3+ and CD8+ T cells in the CT and IM regions, and I4 refers to tumors with high densities of CD3+ and CD8+ in both tumor regions. Furthermore, patients with an Immunoscore > 2 were defined as having a high Immunoscore, while those with an Immunoscore ≤2 were defined as having a low Immunoscore. The densities above the median of each index were categorized as high expression, and those below the median were categorized as low expression.

### Statistical analysis

Statistical analysis was performed with SPSS 24.0 for Windows (SPSS, Chicago, IL, USA). Differences in clinical parameters by the expression levels of CD3+ T cells in the CT and IM, CD8+ T cells in the CT and IM, and the Immunoscore were assessed by a chi-squared test. All values of blood indexes are expressed as the median (minimum-maximum) and are shown in Tables 3, 4, and 5. The distribution of the analyzed parameters was assessed by a nonparametric test. The association between blood indexes and the expression levels of immune cells in the tumor microenvironment was evaluated with two statistical methods to obtain correlation coefficients: Pearson’s correlation for numerical values of both blood indexes and immune cells and Spearman’s correlation for the values of the immune cells and the Immunoscore divided into high or low levels. All analyses were two sided, and a *P* value less than 0.05 was considered statistically significant.

## Results

### Patients’ characteristics

Initially, 1535 CRC patients were found in the clinical database of our center, but only 240 patients with detailed data and well-preserved tumor specimens were finally enrolled in this study. The number of patients with CD8+ T cell expression data in tissue was 240. Due to four patients without successful immunohistochemical staining, the number of patients with CD3+ T cell expression and Immunoscore data was 236. In total, 60.8% of patients were male. The age of patients ranged from 15 to 86 years, with a median age of 65 years. Patients aged 75 or older comprised 14.2% of the population. Most tumors were located on the left side (174, 72.5%). The pathological differentiation in more than half of the tumors was identified as the middle level (159, 66.3%); 77 patients had a low level, and only 4 patients had a high level. Approximately 59.6% (143) of patients were in T3 stage, 30.8% (74) were in T4 stage, 4.6% (11) were in T2 stage, 5.0% (12) were not applicable, and no one was in T1 stage. The patients were almost equally distributed in different N stages, with 73 (30.4%) patients in N0 stage, 71 (29.6%) patients in N1 stage, and 70 (29.2%) patients in N2 stage. Metastases were observed in 191 patients (79.6%). The percentages of cancers by AJCC stage were as follows: 5.8% (14) for stage II cancer, 13.3% (32) for stage III cancer, and 79.6% (191) for stage IV cancer. Most genes associated with treatment choice and prognosis were also included in our study, as shown in Table 1. Microsatellite status was tested in 164 patients; 158 exhibited microsatellite stability, and only 6 exhibited microsatellite instability. KRAS status was determined in 81 patients; 48 harbored wild-type KRAS, and 33 harbored mutation-type KRAS. NRAS status was determined in 47 patients, 46 wild-type and one mutation-type, and HRAS status was determined in 46 patients, all of them were wild-type. BRAF status was available in 60 patients, 59 wild-type and one mutation-type.

**Table 1:** Basic clinicopathological molecular characteristics of 240 colorectal cancer patients

Variable	No. of patients (%)
Sex	
Male	146 (60.8%)
Female	94 (39.2%)
Age	
< 75	206 (85.8%)
≥ 75	34 (14.2%)
Location	
Right	66 (27.5%)
Left	174 (72.5%)
Pathological differentiation	
Poor	77 (32.1%)
Moderate	159 (66.3%)
Well	4 (1.7%)
T stage	
T2	11 (4.6%)
T3	143 (59.6%)
T4	74 (30.8%)
NA	12 (5.0%)
N stage	
N0	73 (30.4%)
N1	71 (29.6%)
N2	70 (29.2%)
NA	26 (10.8%)
M stage	
M0	49 (20.6%)
M1	191 (79.6%)
Stage	
I	0 (0.0%)
II	14 (5.8%)
III	32 (13.3%)
IV	191 (79.6)
MS	
MSS	158 (65.8%)
MSI	6 (2.5%)
NA	76 (31.7%)
KRAS	
Wild-type	48 (20.0%)
Mutation-type	33 (13.7%)
NA	159 (66.3%)
NRAS	
Wild-type	46 (19.2%)
Mutation-type	1 (0.4%)
NA	193 (80.4%)
HRAS	
Wild-type	46 (19.2%)
Mutation-type	0 (0.0%)
NA	194 (80.8%)
BRAF	
Wild-type	59 (24.6%)
Mutation-type	1 (0.4%)
NA	180 (75.0%)

*NA* not applicable

**Fig. 1:**
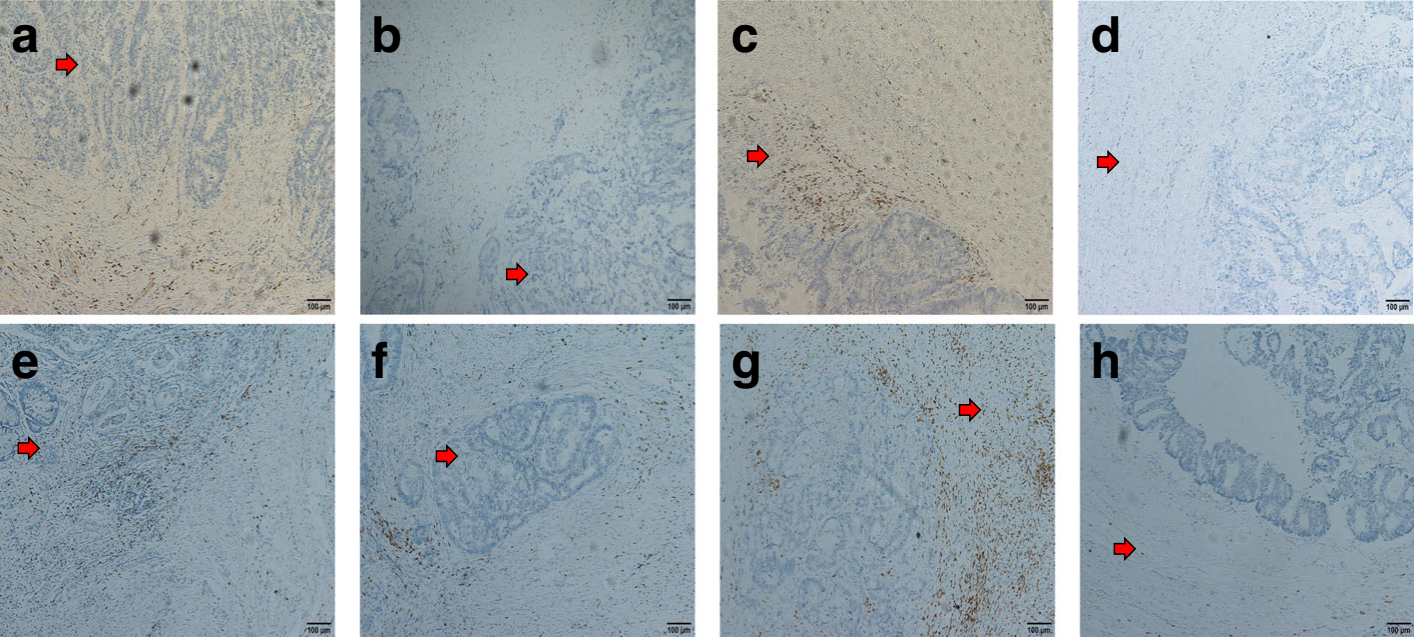
Representative immunohistochemical images of CD3+ and CD8+ T cells in the core of the tumor (CT) and in the invasive margin (IM) of colorectal cancer (200×). **a**, **b** Representative images of high-density and low-density CD3+ T cells in the center of the colorectal cancer; **c**, **d** Representative images of high-density and low-density CD3+ T cells in the invasive margin of the colorectal cancer; **e**, **f** Representative images of high-density and low-density CD8+ T cells in the center of the colorectal cancer; **g**, **h** Representative images of high-density and low-density CD8+ T cells in the invasive margin of the colorectal cancer

The median density of CD3+ T cells in the CT and IM were 1165/mm^2^ (6/mm2–11,917/mm2) and 2107/mm^2^ (25/mm2–15,865/mm2), respectively, and the median number of CD8+ T cells was 96/mm^2^ (2/mm2–4178/mm2) and 262/mm^2^ (1/mm2–2800/mm2), respectively. Low expression was defined as a value below the median, and high expression was defined as a value above the median (Fig. 1).

### Association between basic characteristics and immune cells in the tumor microenvironment

Both the expression of CD3+ (70.6% vs. 29.4%, *P* = 0.015) and CD8+ (67.6% vs. 32.4%, *P* = 0.041) T cells in the IM and Immunoscore (85.3% vs. 14.7%, *P* = 0.011) showed a lower level in older patients (aged 75 years or older). The expression of CD8+ T cells in the IM (67.6% vs. 32.4%, *P* = 0.014) and Immunoscore (78.1% vs. 21.9%, *P* = 0.020) was lower in right-sided tumors than in left-sided tumors. The patients with T4 stage had higher expression of CD8+ T cells in the CT than those with other T stages (62.2% vs. 37.8%, *P* = 0.034). We did not find any relationship between KRAS status and immune cell expression levels in the CT or in the IM; the same was true for the Immunoscore. Also, we did not find any relationship between microsatellite status and immune cell expression levels in the CT or in the IM, or Immunoscore. The above results are shown in Table 2.

**Table 2:** Association between basic characteristics and immune cells in the microenvironment

Variable	*N* = 240	CD3+ T cells in the CT			CD3+ T cells in the IM			CD8+ T cells in the CT			CD8+ T cells in the IM			Immunoscore		
		Low	High	*P*	Low	High	*P*	Low	High	*P*	Low	High	*P*	Low	High	*P*
Sex				0.894			1.000			0.355			0.895			0.326
Male	146	70 (49.3%)	72 (50.7%)		71 (50.0%)	71 (50.0%)		69 (47.3%)	77 (52.7%)		72 (49.3%)	74 (50.7%)		90 (63.4%)	52 (36.6%)	
Female	94	48 (51.1%)	46 (48.9%)		47 (50.0%)	47 (50.0%)		51 (54.3%)	43 (45.7%)		48 (51.1%)	46 (48.9%)		66 (70.2%)	28 (29.8%)	
Age				0.354			0.015			0.355			0.041			0.011
< 75	206	98 (48.5%)	104 (51.5%)		94 (46.5%)	108 (53.3%)		100 (48.5%)	106 (51.5%)		97 (47.1%)	109 (52.9%)		127 (62.9%)	75 (37.1%)	
≥75	34	20 (58.8%)	14 (41.2%)		24 (70.6%)	10 (29.4%)		20 (58.8%)	14 (41.2%)		23 (67.6%)	11 (32.3%)		29 (85.3%)	5 (14.7%)	
Location				0.464			0.661			0.312			0.014			0.020
Right	66	35 (54.7%)	29 (45.3%)		34 (53.1%)	30 (46.9%)		37 (56.1%)	29 (43.9%)		42 (63.6%)	24 (37.4%)		50 (78.1%)	14 (21.9%)	
Left	174	83 (48.3%)	89 (51.7%)		84 (48.8%)	88 (51.2%)		83 (47.7%)	91 (52.3%)		78 (44.8%)	96 (55.2%)		106 (61.6%)	66 (38.4%)	
Pathological differentiation				1.000			1.000			0.580			0.580			0.883
Poor	77	38 (49.4%)	39 (50.6%)		39 (50.6%)	38 (49.4%)		36 (46.8%)	41 (53.2%)		36 (46.8%)	41 (53.2%)		50 (64.9%)	27 (35.1%)	
Moderate and well	163	80 (44.7%)	79 (55.3%)		79 (49.7%)	80 (50.3%)		84 (51.3%)	79 (48.7%)		84 (51.3%)	79 (48.7%)		106 (66.7%)	53 (33.3%)	
T stage				0.480			0.672			0.034			0.572			0.453
T1 + T2 + T3	154	75 (49.7%)	76 (50.3%)		76 (50.3%)	75 (49.7%)		82 (51.9%)	72 (48.1%)		72 (46.8%)	82 (53.2%)		102 (67.5%)	49 (32.5%)	
T4	74	40 (54.8%)	33 (44.6%)		39 (61.9%)	34 (38.1%)		28 (37.8%)	46 (62.2%)		38 (51.4%)	36 (48.6%)		45 (61.6%)	28 (38.4%)	
NA	12															
N stage				0.060			0.080			0.566			0.774			0.221
No	73	31 (43.7%)	40 (56.3%)		32 (45.1%)	39 (54.9%)		32 (43.8%)	41 (56.2%)		32 (43.8%)	41 (56.2%)		42 (59.2%)	29 (40.8%)	
N1 + N2	141	80 (57.6%)	59 (42.4%)		81 (58.2%)	58 (41.8%)		68 (48.2%)	73 (51.8%)		65 (46.1%)	76 (53.9%)		95 (68.3%)	44 (31.7%)	
NA	26															
M stage				0.328			0.102			0.337			0.749			0.606
M0	49	27 (57.4%)	20 (42.6%)		29 (61.7%)	18 (38.3%)		21 (42.9%)	28 (57.1%)		26 (53.1%)	23 (46.9%)		33 (70.2%)	14 (29.88%)	
M1	191	91 (47.6%)	98 (52.4%)		89 (47.1%)	100 (62.9%)		99 (51.8%)	92 (48.2%)		94 (49.2%)	97 (50.8%)		123 (65.1%)	66 (34.9%)	
MS				0.429			1.000			0.682			1.000			1.000
MSS	158	73 (47.4%)	81 (52.6%)		73 (47.4%)	81 (52.6%)		80 (50.6%)	78 (49.4%)		75 (47.5%)	83 (52.5%)		97 (61.4%)	57 (38.6%)	
MSI	6	4 (66.7%)	2 (33.3%)		3 (50%)	3 (50%)		2 (33.3%)	4 (66.7%)		3 (33.3%)	3 (33.3%)		4 (66.7%)	2 (33.3%)	
NA	76															
KRAS				0.161			0.470			0.176			0.262			0.103
W	48	15 (31.9%)	32 (68.1%)		15 (31.9%)	32 (68.1%)		21 (43.8%)	27 (56.2%)		21 (43.8%)	27 (56.2%)		22 (46.8%)	25 (53.2%)	
M	33	15 (48.4%)	16 (51.6%)		15 (45.5%)	18 (64.5%)		20 (60.6%)	13 (39.4%)		19 (57.6%)	14 (42.4%)		21 (67.7%)	10 (32.3%)	
NA	159															

*CT* core of the tumor, *IM* invasive margin, *NA* not applicable

### Association between tumor markers and immune cells in the tumor microenvironment

**Fig. 2:**
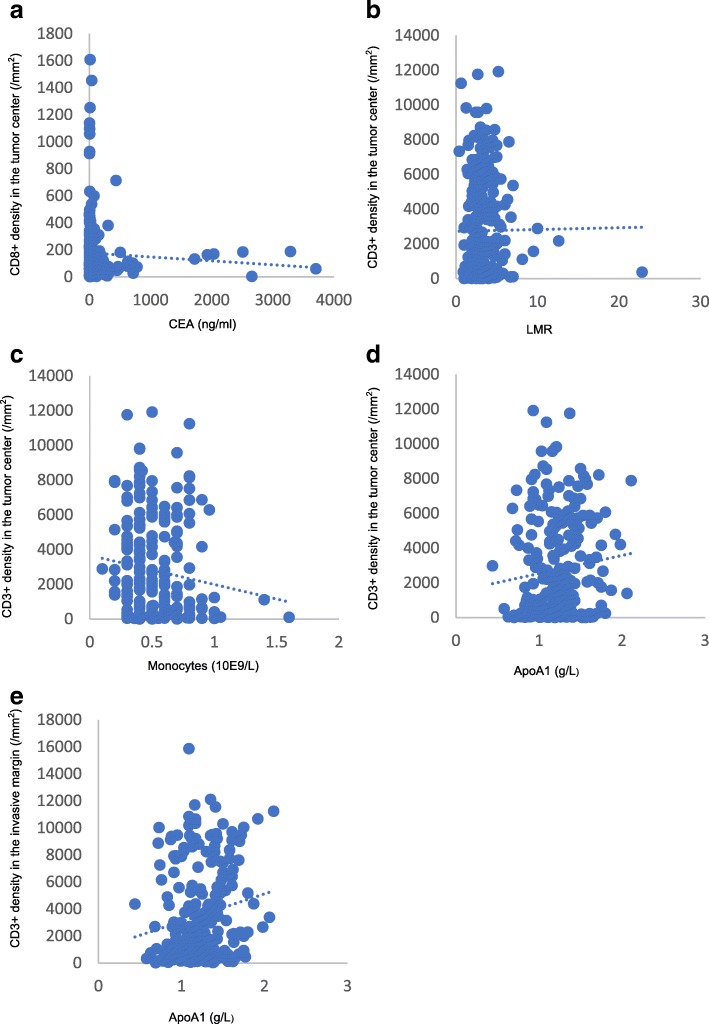
Correlations between the CEA level, number of monocytes, LMR, ApoA1 and density of immune cells in the tumor microenvironment. **a** The density of CD8+ T cells in the core of the tumor showed a tendency that correlated with the level of CEA in the blood, with a coefficient of − 0.135 (*P* = 0.037). **b** The density of CD3+ T cells in the core of the tumor was significantly related to the LMR, with a coefficient of 0.135 (*P* = 0.038). **c** The density of CD3+ T cells in the core of the tumor showed a tendency that correlated with the number of monocytes in the blood, with a coefficient of − 0.127 (*P* = 0.052). **d** The densities of CD3+ T cells in the core of the tumor showed a tendency that correlated with ApoA1, with a coefficient of 0.127 (*P* = 0.051). **e** The densities of CD3+ T cells in the invasive margin were correlated with ApoA1, with a coefficient of 0.169 (*P* = 0.010)

Tumor markers were not significantly associated with CD3+ or CD8+ T cells, as shown in Table 3. However, it showed some trends. The higher the CEA level was in the blood, the lower the expression of CD8+ T cells in the CT (*P* = 0.064). Furthermore, the density of CD8+ T cells in the CT was correlated with the value of CEA, with a coefficient of − 0.135 (*P* = 0.037, Fig. 2a). CA 19–9 did not show any relationship with the infiltration of CD3+ and CD8+ T cells in the CT (*P* = 0.145, *P* = 0.861), IM (*P* = 0.378, *P* = 0.993), or Immunoscore (*P* = 0.544).

**Table 3:** Association between tumor markers and immune cells in the microenvironment

Variable	CD3+ T cells in the CT			CD3+ T cells in the IM			CD8+ T cells in the CT				CD8+ T cells in the IM			Immunoscore		
	Low	High	*P*	Low	High	*P*	Low	High	*P*		Low	High	*P*	Low	High	*P*
CEA	11.00 (0.39–12,257.00)	10.30 (0.56–3704.00)	0.593	12.21 (0.39–12,257.00)	8.53 (0.56–3704.00)	0.120	12.53 (0.56–3704.00)	7.89 (0.39–12,257.00)	0.064		10.54 (0.39–9064)	10.42 (0.56–12,257.00)	0.983	11.34 (0.39–12,257.00)	10.14 (0.56–3704.00)	0.295
CA 19–9	26.01 (0.60–18,707.00)	32.60 (0.60–20,000.00)	0.145	28.29 (0.60–18,707.00)	29.47 (0.60–20,000.00)	0.861	29.97 (0.60–20,000.00)	27.64 (0.60–18,707.00)	0.378		30.35 (0.60–5956.00)	27.64 (0.60–20,000.00)	0.993	28.43 (0.60–18,707.00)	30.33 (0.60–20,000.00)	0.544

*CA 19–9* carbohydrate antigen 19–9, *CEA* carcinoembryonic antigen, *CT* core of the tumor, *IM* invasive margin

### Association between inflammatory markers and immune cells in the tumor microenvironment

As shown in Table 4, the levels of monocytes and the LMR were associated with CD3+ T immune cells in the CT but not in the IM. The more monocytes were in the blood, the lower the CD3+ T cell expression was in the CT (*P* = 0.009). The higher the LMR was, the higher the expression of CD3+ T cells in the CT (*P* = 0.057). Figure 2b presents the statistically significant relationship between the density of CD3+ T cells in the CT and the LMR, with a coefficient of 0.135 (*P* = 0.038). The density of CD3+ T cells in the CT also showed a correlation with the monocyte number in the blood, with a coefficient of − 0.127 (*P* = 0.052, Fig. 2c). We found that the infiltration of CD3+ T cells did not show any relationship with LDH, CRP, ALB, neutrophils, lymphocytes, platelets, NLR, or PLR, either in the CT or in the IM, and no relationship was found between any inflammatory marker and the infiltration of CD8+ T cells in either the CT or the IM.

**Table 4:** Association between inflammatory markers and immune cells in the microenvironment

Variable	CD3+ T cells in the CT			CD3+ T cells in the IM			CD8+ T cells in the CT			CD8+ T cells in the IM			Immunoscore		
	Low	High	*P*	Low	High	*P*	Low	High	*P*	Low	High	*P*	Low	High	*P*
LDH	171.70 (88.40–1957.80)	181.20 (1.97–2461.60)	0.508	177.85 (95.60–1957.80)	174.50 (1.97–2461.60)	0.615	171.05 (1.97–1856.80)	177.05 (88.40–2461.60)	0.427	174.15 (1.97–2461.60)	176.55 (88.40–1957.80)	0.430	174.50 (1.97–1957.80)	175.75 (88.40–2461.60)	0.777
ALB	40.15 (21.20–48.8)	40.25 (28.10–47.20)	0.926	39.75 (30.10–48.80)	40.30 (21.20–47.20)	0.558	40.35 (29.40–48.80)	39.65 (21.20–47.60)	0.402	40.25 (28.30–47.60)	40.10 (21.20–48.80)	0.923	40.45 (30.10–48.80)	39.65 (21.20–47.20)	0.228
CRP	4.80 (0.11–118.70)	4.82 (0.16–157.88)	0.759	4.36 (0.20–156.52)	5.11 (0.11–157.88)	0.582	3.99 (0.11–156.52)	5.12 (0.40–157.88)	0.163	4.40 (0.20–157.88)	4.84 (0.11–156.52)	0.931	4.00 (0.11–156.52)	5.53 (0.16–157.88)	0.164
Neutrophils	3.70 (0.50–14.80)	3.80 (0.90–20.80)	0.956	3.70 (0.50–10.80)	3.85 (0.80–20.80)	0.435	3.80 (0.50–20.80)	3.70 (0.80–14.80)	0.569	3.60 (0.50–11.50)	3.90 (0.90–20.80)	0.341	3.70 (0.50–11.50)	4.05 (0.90–20.80)	0.335
Lymphocytes	1.50 (0.40–13.70)	1.50 (0.20–6.30)	0.850	1.50 (0.40–13.70)	1.50 (0.20–11.40)	0.462	1.60 (0.50–6.30)	1.42 (0.20–13.70)	0.438	1.50 (0.40–13.70)	1.50 (0.20–11.40)	0.894	1.50 (0.40–13.70)	1.40 (0.20–11.40)	0.452
Monocytes	0.50 (0.20–1.60)	0.50 (0.10–0.96)	0.009	0.50 (0.10–1.60)	0.50 (0.20–1.40)	0.151	0.50 (0.20–1.05)	0.50 (0.10–1.60)	0.335	0.50 (0.20–1.00)	0.50 (0.10–1.60)	0.838	0.50 (0.20–1.60)	0.50 (0.10–1.40)	0.450
Platelets	238.00 (45.00–615.00)	244.50 (10.30–1181.00)	0.796	231.00 (45.00–1181.00)	249.00 (10.30–1060.00)	0.328	245.50 (79.00–1181.00)	230.50 (10.30–594.00)	0.310	235.00 (79.00–1060.00)	247.50 (10.30–1181.00)	0.169	241.50 (45.00–1181.00)	241.00 (10.30–594.00)	0.743
NLR	2.38 (0.21–16.75)	2.60 (0.43–62.50)	0.285	2.41 (0.21–16.75)	2.55 (0.43–62.50)	0.629	2.57 (0.43–16.00)	2.47 (0.21–62.50)	0.644	2.26 (0.21–16.75)	2.53 (0.43–62.50)	0.269	2.41 (0.21–16.75)	2.61 (0.43–62.50)	0.156
PLR	156.13 (7.58–541.70)	165.48 (7.92–984.17)	0.398	159.41 (7.58–984.17)	163.09 (7.92–581.11)	0.412	158.64 (25.56–984.17)	168.98 (7.58–530.00)	0.964	162.24 (7.58–530.00)	165.40 (7.92–984.17)	0.277	159.03 (7.58–984.17)	165.56 (7.92–581.11)	0.297
LMR	2.80 (0.90–22.83)	3.08 (0.40–12.60)	0.057	2.82 (0.90–22.83)	3.00 (0.40–9.50)	0.157	3.00 (1.00–12.60)	2.81 (0.40–22.83)	0.346	2.88 (0.80–22.83)	3.00 (0.40–10.00)	0.750	2.85 (0.90–22.83)	3.00 (0.40–10.00)	0.5853

*ALB* albumin, *CRP* C-reactive protein, *CT* core of the tumor; *IM* invasive margin, *LDH* lactate dehydrogenase, *LMR* lymphocyte-to-monocyte ratio, *NLR* neutrophil-to-lymphocyte ratio, *PLR* platelet-to-lymphocyte ratio

### Association between lipid metabolism and immune cells in the tumor microenvironment

Among all the lipid metabolism markers examined, ApoA1 was the only marker associated with immune cells, as shown in Table 5. ApoA1 was associated with the expression of CD3+ T cells regardless of the location (CT (*P* = 0.022) and IM (*P* = 0.002)), and the higher ApoA1 was in the blood, the higher the expression of CD3+ T cells. In addition, the densities of CD3+ T cells in both the CT and IM were correlated with ApoA1, with coefficients of 0.127 (*P* = 0.051) and 0.169 (*P* = 0.010), respectively, as shown in Fig. 2d and e.

**Table 5:** Association between lipid metabolism markers and immune cells in the microenvironment

Variable	CD3+ T cells in the CT			CD3+ T cells in the IM			CD8+ T cells in the CT				CD8+ T cells in the IM			Immunoscore		
	Low	High	*P*	Low	High	*P*	Low	High	*P*		Low	High	*P*	Low	High	*P*
CHO	4.97 (1.91–15.30)	4.90 (1.97–10.28)	0.801	5.00 (1.91–15.30)	4.82 (1.97–10.28)	0.487	4.88 (2.92–15.30)	4.88 (1.91–14.97)	0.796		4.93 (1.97–10.28)	4.86 (1.91–15.30)	0.990	4.98 (1.91–15.30)	4.80 (1.97–10.28)	0.386
TG	1.08 (0.44–4.83)	1.15 (0.52–5.82)	0.959	1.12 (0.44–4.83)	1.13 (0.52–5.82)	0.797	1.07 (0.53–4.92)	1.14 (0.44–5.82)	0.386		1.13 (0.52–5.82)	1.10 (0.44–3.76)	0.365	1.09 (0.44–4.92)	1.13 (0.52–5.82)	0.986
HDL-C	1.13 (0.59–2.73)	1.18 (0.30–2.19)	0.475	1.18 (0.59–2.73)	1.14 (0.30–2.17)	0.410	1.18 (0.68–2.73)	1.13 (0.30–2.43)	0.364		1.14 (0.30–2.19)	1.17 (0.59–2.73)	0.627	1.18 (0.59–2.73)	1.14 (0.30–2.17)	0.679
LDL-C	3.06 (0.77–13.48)	3.10 (0.80–126.10)	0.758	3.04 (0.77–13.48)	3.12 (0.80–126.10)	0.861	3.05 (0.77–126.10)	3.01 (0.80–13.48)	0.819		3.13 (0.80–126.10)	3.00 (0.77–13.48)	0.735	3.13 (0.77–126.10)	3.00 (0.80–8.56)	0.394
ApoA1	1.17 (0.58–2.06)	1.24 (0.44–2.11)	0.022	1.18 (0.58–1.77)	1.26 (0.44–2.11)	0.002	1.21 (0.58–2.11)	1.18 (0.44–2.06)	0.192		1.20 (0.44–2.06)	1.19 (0.63–2.11)	0.498	1.19 (0.58–2.06)	1.22 (0.44–2.11)	0.280
ApoB	0.97 (0.32–2.80)	0.98 (0.39–2.08)	0.828	0.97 (0.32–2.80)	0.97 (0.44–2.11)	0.929	0.97 (0.39–1.69)	0.97 (0.32–2.80)	0.408		0.97 (0.39–2.08)	0.97 (0.32–2.80)	0.832	0.98 (0.32–2.80)	0.96 (0.39–2.08)	0.801

*ApoA1* apolipoprotein A1, *ApoB* apolipoprotein B, *CHO* cholesterol, *CT* core of the tumor, *HDL* high-density lipoprotein, *IM* invasive margin, *LDL* low-density lipoprotein, *TG* triglyceride

## Discussion

The present study investigated the relationship between clinicopathologic characteristics and blood indexes with CD3+ and CD8+ T cells in the CRC tissue microenvironment. We observed that older patients had low expression of CD3+ and CD8+ T cells in the IM and a low Immunoscore. The expression of CD8+ T cells in the IM and the Immunoscore were lower in right-sided tumors than in left-sided tumors. The higher expression of CD8+ T cells in CT was found in the group of patients in T4 stage. The higher the CEA level in the blood, the fewer CD8+ T cells were in the CT. Either fewer monocytes or a higher LMR in the blood, the larger number of CD3+ T cells in the CT. The higher ApoA1 was in the blood, the more CD3+ T cells were in the CT and in the IM. The results from this study suggest that noninvasive peripheral blood analysis of some markers could be distinctly helpful in assessing the immunity status in the tumor microenvironment.

Elder patients had different immunity from the younger patients. We observed fewer T cells in the tumor tissue of elderly patients than in that of younger patients. When the patient’s age was older than 75 years, both the CD3+/CD8+ T cells in the IM and the Immunoscore became less statistically significant. At the same time, the CD3+/CD8+ T cells also decreased in the CT despite no significant difference. Aging resulted in deteriorating health and an increased risk of cancer accompanied by progressive, multidimensional, physiological degeneration with an immune system decrease thought to play a key role in regulating these declines [16], which is known as immunosenescence [17]. Additionally, the tumor immuno-microenvironment may be altered during aging as a result of age-related immune dysfunction. Provinciali et al. found that mammary tumors in elderly mice had reduced numbers of infiltrating CD3+ and CD8+ T cells compared to younger mice [18]. Many reasons could explain why T cells may be significantly decreased in older patients. Thymic output decreases with age, resulting in lower proportions of T cell populations, contributing to an inability to mount T cell responses to novel tumor-associated antigens [19]. Moreover, T lymphocytes from older individuals present a significant reduction in the activation of nuclear factor-kΒ, which is responsible for the expression of proinflammatory cytokine genes [20]. Thus, T cells would become deactivated in older people. This study found 34 patients over 75 years old with decreased expression of CD3+/CD8+ T cells in CRC tissue compared to the 206 younger patients. It would be interesting to further investigate this change in large number of older patients with CRC or other cancers.

The data from this study revealed that right- and left-sided CRCs had obviously different Immunoscores and T cell concentrations in cancer tissue. The CD8+ T cells in the IM and the Immunoscore were both low for right-sided tumors. CD8+ T cells are cytotoxic T cells and play a central role in anticancer immunity; therefore, our results could be useful for explaining the poor prognosis in right-sided CRC. Recently, Jonna Bernstsson et al. reported that CD8+ T cell infiltration differed significantly according to the tumor side in CRC, with denser collection in a left-sided tumor than in a right-sided tumor [21], which is in line with our result. Many studies suggest differences, including epidemiology, tumor characteristics and prognosis, between right-sided and left-sided CRCs [22]. Moreover, right-sided tumors demonstrate diverse genetic and molecular characteristics compared to left-sided tumors [23]. These differences in biological behavior may induce different responses to chemotherapy and targeted agents [24]. In the present study, we found a difference in the immuno-microenvironment between proximal and distant CRCs, which provides further evidence that anatomical subsites may represent distinct disease entities.

Interestingly, we observed a higher density of CD8+ T cells in the CT in T4 stage. T4 stage indicates that tumor cells invade through the visceral peritoneum or invade or adhere to adjacent organs or structures. We assume that it would expose more antigens that could lead to inflammation. Cancer is closely related to inflammation. Many cancers arise from sites of infection, chronic irritation, and inflammation. We speculated that T4 tumors might induced obvious inflammation and then attracted more immune cells. Whether those patients are more likely to benefit from immunotherapy is another appealing topic since T4 has more CD8+ T cells in the CT.

The present study elucidated that the immune concentration in cancer tissue is closely related to inflammation. The more CD3+ T cells were in the CT, the fewer monocytes and the higher LMR were in the blood. Monocytes and their progeny within the tumor microenvironment can produce factors promoting the growth, migration, invasion, and survival of tumor cells [25]. Nevertheless, another study showed that a dense number of macrophages in the blood indicated a good prognosis for CRC patients [26]. Tumor-associated macrophages (TAMs), which are derived from circulating monocyte populations, play a key role in the tumor immuno-microenvironment, encouraging metastasis and tumor progression [27]. The association between systemic inflammation and the poor prognosis of cancers could be explained by the effects on the tumor immuno-microenvironment. It has already been shown that the levels of tumor-infiltrating lymphocytes predict better survival in CRC patients [28]. However, little mechanistic evidence linking the poor prognosis of cancer patients with systemic inflammation exists, and hardly any study has focused on the relationship between the immuno-microenvironment in tumor tissue and systemic inflammation. Our study performed a preliminary exploration and found that monocytes and the LMR are significantly connected with the number of immunity cells in CRC tissue.

We observed that the higher the CEA level in the blood, the fewer CD8+ T cells were in the CT. CEA is the most commonly used tumor marker in patients with CRC. CEA is implicated in cell adhesion, cell-to-cell interactions and signal transduction in cancer cells [29]. CRC patients with abnormal CEA values have been shown to have lower overall survival [30]. It is still unknown how circulating CEA, released by CRC cells, inhibits the migration of CD8+ T cells to the tumor center, which is valuable for studying intensive molecular mechanisms.

The present study demonstrated that the higher the circulating ApoA1 level, the more CD3+ T cells were in both the CT and the IM. ApoA1, a predominant protein component in HDL, transports excess CHO from peripheral tissues to the liver and has anti-inflammatory, antiapoptotic and antioxidant functions [31]. Studies have confirmed that ApoA1 could alter TAMs from a protumor M2 to an antitumor M1 phenotype [32]. It also modulates regulatory T cells [33]. Thus, ApoA1 is situated at the nexus of a number of physiologically significant immune functions. Furthermore, decreased serum ApoA1 levels have been reported to correlate with poor CRC outcomes [15]. We inferred that CD3+ T cells aggregated in both the CT and the IM for CRC with high ApoA1, consequently, ApoA1 was strongly positively correlated with CRC patient survival.

Cancer immunotherapies that inhibit negative immune feedback, such as those targeting programmed cell death 1 (PD1)/programmed cell death-ligand 1 (PDL1) and cytotoxic T-lymphocyte-associated protein 4 (CTLA4), have proven efficacious against several tumor types [34, 35]. However, not all cancer patients benefit from immunotherapies, and to date, PD1/PDL1 have been approved in CRC only with MSI-H by the Food and Drug Administration [36]. No other definitive biomarker exists to easily predict the outcomes of this immune system activity. Immune infiltration of cancers has been suspected to be a positive factor for patient outcome since the early 1900s [37]. However, these immune cells do not have a major classification for clinical decision making. Franck Pagès et al. confirmed that the Immunoscore had the highest relative contribution to the risk of all clinical parameters, including the AJCC/UICC TNM classification system [8]. The Immunoscore represents a standardized immune-based assay for the classification of cancer. On the basis of our identification of an association between markers in the blood and the densities of immune cells in the tumor microenvironment, we proposed that the CEA level, number of monocytes, LMR, and ApoA1 may be used to ascertain the immunological status in the tumor microenvironment. Importantly, the CEA level, number of monocytes, LMR, and ApoA1 could be easily calculated in the blood, eliminating the need for invasive procedures and complex processes to evaluate the tumor immuno-microenvironment.

Nevertheless, this study has a few limitations. First, as a retrospective study, it is still possible that patients had potential systemic inflammation not related to cancer. Second, we did not analyze the specific subtypes of T cells excluding CD3+ and CD8+ immune cells, although there are different roles and prognoses that other immune cells may play in the tumor microenvironment. Third, the underlying reason explaining why blood markers were associated with the density of CD3+ and CD8+ immune cells requires further investigation.

## Conclusions

Our results demonstrate that age, T stage, tumor location, CEA level, number of monocytes, LMR and ApoA1 are associated with immune cell densities in the tumor microenvironment. This study suggests the possibility that the presence of cancer-changed immune cells in the tumor microenvironment could be evaluated noninvasively with markers in peripheral blood samples and clinicopathologic characteristics. In the next step, it will be significant to establish a nomogram model including these indexes to predict the immunity status in the tumor microenvironment, and to explore the predictive and prognostic role of Immunoscore in CRC. It is also attractive to explore why these blood markers are associated with the density of CD3+ and CD8+ immune cells in tumors.

## Data Availability

All data generated that are relevant to the results presented in this article are included in this article. Other data that were not relevant for the results presented here are available from the corresponding author upon reasonable request.

## Abbreviations

AJCC: The American Joint Committee on Cancer
ALB: Albumin
ApoA1: Apolipoprotein A1
ApoB: Apolipoprotein B
CA 19–9: Carbohydrate antigen 19–9
CEA: Carcinoembryonic antigen
CHO: Cholesterol
CRC: Colorectal cancer
CRP: C-reactive protein
CT: Core of the tumor
HDL: High-density lipoprotein
IM: Invasive margin
LDH: Lactate dehydrogenase
LDL: Low-density lipoprotein
LMR: Lymphocyte-to-monocyte ratio
NLR: Neutrophil-to-lymphocyte ratio
PLR: Platelet-to-lymphocyte ratio
TG: Triglyceride
UICC: Union for International Cancer Control
